# ΔNp63α drives serine synthesis to promote carboplatin resistance in NSCLC

**DOI:** 10.1038/s41419-026-08497-4

**Published:** 2026-02-17

**Authors:** Liyuan Deng, Xin Yang, Junli Zhang, Xuanyu Zhou, Ruidong Ma, Zhiqiang Wu, Hu Chen

**Affiliations:** 1 Department of Cardiothoracic Surgery, School of Clinical Medicine and The First Affiliated Hospital of Chengdu Medical College, Chengdu, China; 2 Department of Pediatrics, School of Clinical Medicine and The First Affiliated Hospital of Chengdu Medical College, Chengdu, China; 3 Department of Pathology, School of Clinical Medicine and The First Affiliated Hospital of Chengdu Medical College, Chengdu, China; 4 Key Laboratory of Geriatric Respiratory Diseases of Sichuan Higher Education Institute, Chengdu, China

**Keywords:** Non-small-cell lung cancer, Non-small-cell lung cancer

## Abstract

Serine metabolism is a critical vulnerability in cancer; however, its role in mediating therapeutic resistance in non-small cell lung cancer (NSCLC) remains incompletely understood. In this study, we identify key enzymes in the serine synthesis pathway (SSP), namely PHGDH, PSAT1 and PSPH, as well as the serine transporter SLC1A4, which are significantly overexpressed in lung cancer and correlate with poor patient prognosis. We show that serine contributes to carboplatin resistance in NSCLC, particularly in lung squamous cell carcinoma (LUSC). Notably, the LUSC lineage-specific oncogene ΔNp63α serves as a master transcriptional regulator of serine biosynthesis, directly transactivating the expression of PHGDH, PSAT1, PSPH, and SLC1A4. ΔNp63α-driven serine biosynthesis supports nucleotide synthesis and enhances antioxidant defense, enabling cancer cells to survive carboplatin-induced DNA damage and oxidative stress, thereby promoting therapeutic resistance. The combined inhibition of endogenous serine synthesis and restriction of exogenous serine/glycine significantly overcomes ΔNp63α-mediated carboplatin resistance. Our findings establish the ΔNp63α-SSP axis as a critical mechanism driving carboplatin resistance in LUSC. These results highlight dual-targeted disruption of serine availability as a promising therapeutic strategy to overcome chemotherapy resistance in ΔNp63α-driven LUSC. This study underscores the importance of lineage-specific metabolic dependencies as essential targets for precision oncology in NSCLC.

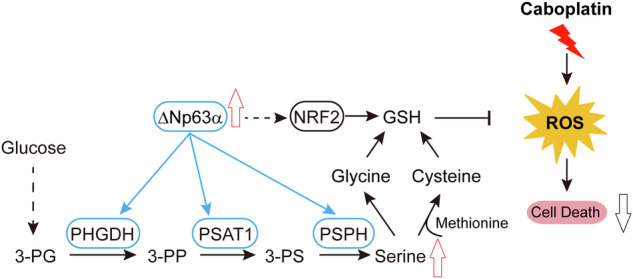

## Introduction

Lung cancer remains the leading cause of global cancer-related mortality, accounting for over 1.5 million annual deaths annually [1, 2]. Non-small cell lung cancer (NSCLC) accounting for approximately 85% of cases, with adenocarcinoma (LUAD) and squamous cell carcinoma (LUSC) being the predominant histological subtypes [3]. While LUAD has benefited from targeted therapies against driver alterations (e.g., EGFR mutations; ALK, ROS1, RET, MET, BRAF fusions/rearrangements) and antibody-based regimens [4], therapeutic options for LUSC remain limited. LUSC patients often have fewer actionable targets and historically rely on platinum-doublet chemotherapy (e.g., carboplatin-based regimens). Recent approvals include EGFR monoclonal antibodies (e.g., Necitumumab) [5] and immune checkpoint inhibitors (e.g., Pembrolizumab, Tislelizumab, Sintilimab) [6–8], while platinum-immunotherapy combinations have now become the standard first-line treatment for metastatic disease [9, 10]. Despite these advancements, durable responses and survival benefits remain limited [11], highlighting the urgent need to elucidate resistance mechanisms specific to LUSC.

Cancer cells reprogram their metabolism to sustain proliferation and survival, creating targetable vulnerabilities [12]. Serine metabolism has emerged as a critical hub in this metabolic reprogramming [13]. Serine and its derivative glycine (via SHMT1/2) fuel nucleotide, protein, and lipid biosynthesis, antioxidant defenses (including glutathione and NADPH production), and one-carbon metabolism for methylation reactions [14]. Consequently, serine dependency in cancer has spurred the development of antifolates (e.g., methotrexate) [15], targeting dihydrofolate reductase (DHFR), thymidylate synthase (TYMS), and glycinamide ribonucleotide formyltransferase (GART) [16]. Although serine is considered a non-essential amino acid, it is avidly consumed by tumors [17, 18]. Cells adapt to serine deprivation via the de novo serine synthesis pathway (SSP), which diverts glycolytic intermediates (e.g., 3-phosphoglycerate) into serine biosynthesis under PKM2 inhibition [19, 20]. This adaptation is orchestrated by ATF4 and the histone methyltransferase G9a, which transcriptionally upregulate SSP enzymes [20–22]. SSP efficacy varies with genetic context: PHGDH amplification or overexpression desensitizes cells to serine restriction [23, 24]. while oncogenes (KRAS, MYC, MDM2, NRF2) enhance SSP enzyme expression and confer resistance [21, 25–27]. Conversely, p53 represses PHGDH [28], though p53 loss paradoxically increases oxidative stress vulnerability during SSP activation [17].

Dietary serine restriction has emerged as a promising therapeutic strategy, with multiple studies demonstrating its efficacy in inhibiting tumor growth in vivo [17, 25, 29–31]. Mice fed serine/glycine-free diets exhibit reduced circulating serine and glycine levels, alongside suppressed tumor progression in xenograft and genetically engineered models. As predicted by in vitro studies, the anti-tumor effect of dietary restriction is potentiated by co-targeting antioxidant defense mechanisms [25]. Conversely, tumors with KRAS activation or PHGDH amplification or overexpression show enhanced de novo serine synthesis, reducing their sensitivity to dietary restriction [25, 30]. This resistance highlights the potential of combining SSP inhibition with dietary serine depletion to improve therapeutic outcomes, as demonstrated by recent work showing synergy between PSAT1 deletion and serine restriction in suppressing MYC-driven liver cancer [32].

**Fig. 1:**
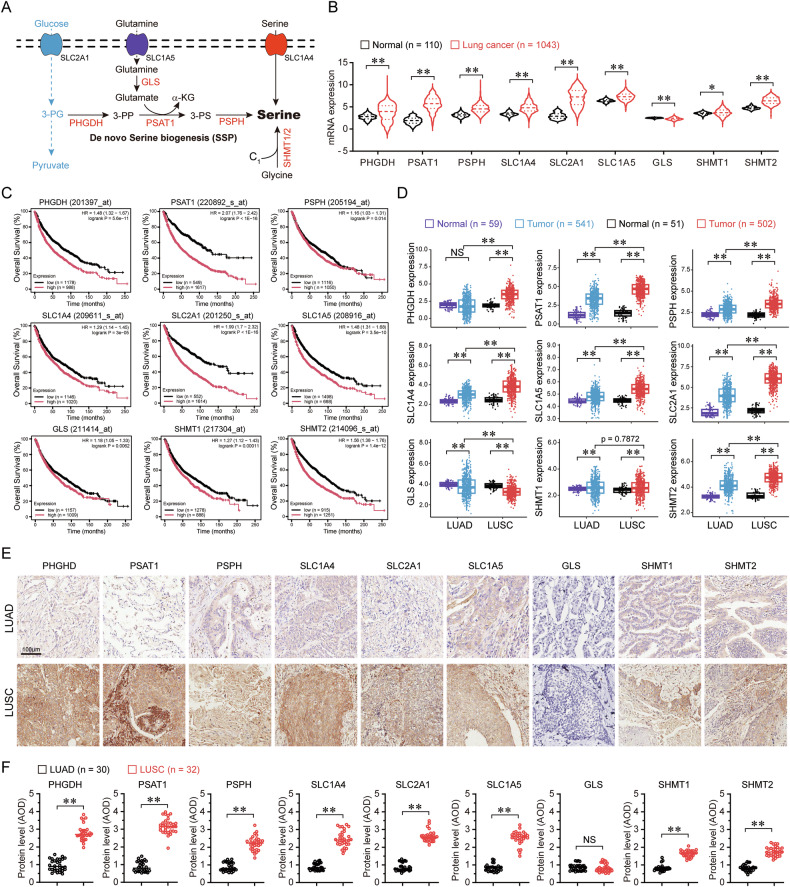
Elevated serine synthesis enzymes are associated with poor prognosis in lung cancer. **A** Serine can be either taken up from the environment or synthesized de novo through the serine synthesis pathway (SSP). The SSP consists of a three-step enzymatic process, starting with the glycolytic intermediate 3-phosphoglycerate (3-PG) which is converted to 3-phosphohydroxypyruvate (3-PP) by phosphoglycerate dehydrogenase (PHGDH). The 3-PP is then converted to 3-phosphoserine (3-PS) by phosphoserine aminotransferase 1 (PSAT1) through a glutamate-dependent transamination reaction. Finally, phosphoserine phosphatase (PSPH) catalyzes the hydrolysis of 3-PS to produce serine. Glycine is a precursor of nucleotides and amino acids. **B** mRNA expression levels of serine metabolism enzymes in lung cancer versus normal tissue, analyzed from the TCGA Firehose Legacy dataset on the cBioPortal platform. **C** Overall survival curves for lung cancer stratified by the mRNA expression levels of serine metabolism enzymes, as analyzed using the Kaplan–Meier Plotter. **D** mRNA expression levels of serine metabolism enzymes in LUAD and LUSC compared to normal tissue, as analyzed from the TCGA Firehose Legacy dataset on the cBioPortal platform. **E** Representative microphotographs showing the immunoreactivity of serine metabolism enzymes in tissue microarrays of LUAD and LUSC. **F** Quantitative analysis of serine metabolism enzyme expression in tissue microarrays of LUAD and LUSC. *p < 0.05; **p < 0.01; NS not significant.

Metabolic reprogramming, a hallmark of malignancy, represents a rich source of therapeutic vulnerabilities, enabling cancer cells to sustain proliferation, evade stress, and develop therapy resistance [12, 33]. Oncogenic drivers and tumor suppressor losses orchestrate this reprogramming by modulating the expression and activity of metabolic enzymes, creating targetable dependencies [34]. Unlike normal cells, which rely on oxidative phosphorylation, most cancers exhibit the “Warburg effect”, favoring aerobic glycolysis despite its low ATP yield (2 mol ATP/mol glucose) [35]. This metabolic shift facilitates carbon influx for biomass accumulation, diverting glycolytic intermediates, such as 3-phosphoglycerate, into serine biosynthesis via a three-step enzymatic cascade (Fig. 1A). Serine is critical for cancer progression, fueling macromolecule synthesis (proteins, lipids, nucleotides), antioxidant defense (glutathione production), and one-carbon metabolism (methylation, tRNA formylation) [13]. As a natural allosteric activator of PKM2, the final enzyme in glycolysis, serine also modulates glycolytic flux [19]. Consequently, targeting serine availability (via SSP inhibition or uptake blockade) enhances therapeutic efficacy [25, 31, 36].

Despite its established role in cancer metabolism, the contribution of serine to carboplatin resistance in NSCLC remains poorly defined. Here, we demonstrate that serine synthesis enzymes are overexpressed in lung cancer, particularly in LUSC, and this overexpression correlates with poor prognosis. Serine promotes carboplatin resistance by scavenging reactive oxygen species (ROS) and attenuating DNA damage. ΔNp63α, a master regulator of epidermal differentiation and lineage-specific oncogene in SCC, transcriptionally activates SSP enzymes (PHGDH, PSAT1, PSPH, SLC1A4). Co-inhibition of SSP and restriction of exogenous serine effectively overcomes ΔNp63α-mediated carboplatin resistance. Our findings identify SSP co-targeting as a novel strategy to combat carboplatin resistance in NSCLC, with relevance for the therapeutically challenging LUSC subtype.

## Materials and methods

### Patients and ethics statement

18 patients diagnosed as having lung adenocarcinoma and 21 patients diagnosed as having squamous cell carcinoma were enrolled in this study at Department of cardiothoracic surgery, the First Affiliated Hospital of Chengdu Medical College. Tumors tissues were obtained during surgery and used for serine content analysis. Written informed consent was obtained from the lung cancer patient. This study protocol was approved by the Institutional Review Board of the First Affiliated Hospital of Chengdu Medical College (approval No. 2022CYFYIRB-BA-Aug12).

### Cell culture and reagents

The human lung cancer cell lines (PC9, NCI-H1975, NCI-H520, NCI-H1703 and HCC95) and human bronchial epithelial cells (BEAS-2B) were obtained from American Type Culture Collection (ATCC). All cell lines were verified through short tandem repeat (STR) DNA profiling. Mycoplasma contamination was tested and found to be negative. HEK-293T cells were cultured in DMEM (Gibco, Thermo Fisher Scientific, Shanghai, China) supplemented with 10% fetal bovine serum (FBS) (Cat. No. 10099158, Gibco). PC9, NCI-H1975, NCI-H520, NCI-H1703 and HCC95 cells were cultured in RPMI-1640 (Gibco) supplemented with 10% FBS (Gibco). BEAS-2B cells were cultured in BEGM kit (Cat. No. CC-3170, Lonza Bioscience). The media without serine (-S), or without serine and glycine (-SG) were custom-formulated from Gibco on the base of RPMI-1640 medium. To eliminate the confounding effects of exogenous serine and glycine present in standard fetal bovine serum (FBS), all amino acid starvation experiments were conducted using custom-formulated media supplemented with dialyzed FBS (Cat. No. 30067334, Gibco). All cells were cultured at 37 °C in a humidified incubator with 5% CO_2_. Carboplatin, N-acetylcysteine (NAC), formate, and NCT-503 were purchased from Selleck (Selleck, Shanghai, China).

### Plasmid constructs

The short hairpin RNAs (shRNAs) targeting human p63 or ATF4 was generated by inserting specific oligoes into a pLKO.1-puromycin lentiviral vector (10878, Addgene, Cambridge, MA, USA). The pLKO.1-scramble vector (#1864, Addgene) was used as a negative control vector and contained a scrambled shRNA (shC). The oligoes used in this study were listed below. shp63 #1: GAGTGGAATGACTTCAACTTT, shp63 #2: CATCTGACCTGGCATCTAATT; shATF4 #1: GTCCTCCACTCCAGATCATTC, shATF4 #2: GCCTAGGTCTCTTAGATGATT. The constructs encoding human ΔNp63α, ΔNp63α^R304W^, TAp63α, ΔNp63β ΔNp63γ, ATF4 or NRF2 were described in previous study [37–39].

For the promoter assay, the fragment (−3000 bp to +1000 bp) of human PHGDH, PSAT1, PSPH and SLC1A4 promoters, containing ΔNp63α putative binding sites, was inserted into the Gluc-On promoter reporter vector (pEZX-PG04, GeneCopoeia, Guangzhou, China) and designated as PHGDH-Gluc, PSAT1-Gluc, PSPH-Gluc, SLC1A4-Gluc, respectively.

### Lentiviral packaging and infection

Recombinant lentivirus particles were generated as described previously [37]. Cells were infected with lentivirus at 50% confluence in the presence of 10 μg/mL polybrene for 24 h. At 48 h post-infection, stable cells were selected by treatment with 2 μg/mL puromycin (A1113803, Gibco) or 10 μg/mL blasticidin (A1113903, Gibco).

### Cell viability assay

For the cell viability assays, cells were plated in a 96-well plate at a density of 2 × 10^4^/well and treated with carboplatin. CCK-8 solution (C0039, Beyotime Biotechnology, Shanghai, China) was added to each well and incubated for 2 h, after which the absorbance was measured at 450 nm.

### Intracellular serine, GSH, formate assay

The serine levels were measured using the serine assay kit (ab241027, abcam, Shanghai, China). The GSH levels were evaluated using the GSH assay kit (S0053, Beyotime Biotechnology), and formate levels were monitored using the formate assay kit (ab111748, abcam). Additionally, the protein concentration of cellular samples was assayed using BCA protein assay kit (P0010S, Beyotime Biotechnology).

### Reactive oxygen species (ROS) measurement

ROS levels in cells were determined as described previously [39]. Briefly, after treatment with carboplatin, cells were incubated with DCFH-DA at 37 °C, and ROS-mediated oxidation of the fluorescent compound DCF was measured using a Reactive Oxygen Species Assay Kit (S0033s, Beyotime Biotechnology) according to the manufacturer’s instructions. Fluorescence of oxidized DCF was measured at an excitation wavelength of 488 nm and an emission wavelength of 535 nm using a FACS scan flow cytometer (FACS Calibur, BD Biosciences).

### Quantitative RT-PCR

Quantitative RT-PCR (QPCR) was used to detect the mRNA level and was performed as described previously [37]. QPCR primer sequences are listed below. GAPDH-F: AAGGTGAAGGTCGGAGTCAA, GAPDH-R: AATGAAGGGGTCATTGATGG; ΔNp63-F: GGAAAACAATGCCCAGACTC, ΔNp63-R: CTGCGCGTGGTCTGTGTTAT; PHGDH-F: AGGGAGAGAAAATCCACATTCTTGG, PHGDH-R: GGCTCTTTATTAGACGGTTATTGCT; PSAT1-F: GGGATGGCTACAAAAAGTTAACACA, PSAT1-R: CAGCTTTACAGAGTTGTGGATGTTT; PSPH-F: TGAATCTGGTGGAAAAGGAAAAGTG, PSPH-R: ATTTGGCGTTATCCTTGACTTGTTG; SLC1A4-F: GTCTATCTTCCTGGTTTTCAGTCCA, SLC1A4-R: GTTAGAAAGAGAACAGCAGGGGAAA.

### Immunoblot analysis

Immunoblot analysis was used to detect protein level and was performed as described previously [37]. Antibodies for PHGDH (Cat. No. 14719-1-AP, 1:1000), PSAT1 (Cat. No. 10501-1-AP, 1:1000), PSPH (Cat. No. 14513-1-AP, 1:1000), SLC1A4 (Cat. No. 13067-2-AP, 1:1000), actin (Cat. No. 66009-1-Ig, 1:5000), were purchased from Proteintech (Wuhan, Hubei, China). Antibody for γH2AX (#9718, 1:1000) was purchased from Cell Signaling Technology (Danvers, MA, USA). Antibody for p63 (Cat. No. 381215, 1:1000) was purchased from ZEN-Bioscience (Chengdu, Sichuan, China).

### Dual-luciferase reporter assay

Luciferase reporter assays were performed using the Secrete-Pair^TM^ Dual Luminescence Assay Kit (GeneCopoeia, USA) according to the manufacturer’s instructions. For gene promoter analyses, cells were co-transfected with 500 ng of reporter plasmids (PHGDH-Gluc, PSAT1-Gluc, PSPH-Gluc, or SLC1A4-Gluc) and 750 ng of ΔNp63α expression plasmids (ΔNp63α^WT^ or ΔNp63α^R304W^) or empty vector (EV). 48 h post-transfection, cell culture media were collected, and Gluc and SEAP activities were measured. The Gluc activity was normalized to SEAP activity.

### Chromatin immunoprecipitation (ChIP) assay

The JASPAR database (http://jaspar.genereg.net/) was used to predict the potential transcription factor binding sites on the enhancer or promoter. Based on the predicted results from JASPAR, ChIP assays were conducted to confirm the binding region of ΔNp63α on gene enhancer or promoter in HCC95 cells using the ChIP-IT Kit (53009, Active Motif, USA). The antibodies specific for ΔNp63 (619502, Biolegend, 1:50) or normal rabbit IgG (#2927, CST, 1:50) were used, as described previously [40]. Immunoprecipitated DNA was then subjected to PCR to amplify fragments of gene enhancer or promoter elements using the indicated primers listed below, PHGDH-ChIP-F: GCACAACTTCGCCCAGCCGT, PHGDH-ChIP-R: GCGGTGCGGGGCTCGGGCCG; PSAT1-ChIP-F: CACCTTCTTCTGGTTTGGGC, PSAT1-ChIP-R: CAGGCAAGTCCTTTAAAGTC; PSPH-ChIP-F: CAGCTGGGCACGGGCCTGGT, PSPH-ChIP-R: GGATTACAGGCTCGCGCCAC; SLC1A4-ChIP-F: AGAGGAGCAGGCGCCCTTG, SLC1A4-ChIP-R: CCTCCTGGTCCCCATGGTTC.

### CDX tumors and drug sensitivity assay

Animal experiments were conducted in accordance with Guidelines for Animal Experiments at Chengdu Medical College, with approval from the Institutional Animal Care and Use Committee. Athymic nude mice purchased from GemPharmatech (Nanjing, China). To investigate the role of serine in carboplatin-mediated antitumor effect in vivo, mice were injected at 8 weeks of age with 5 × 10^5^ NCI-H520 cells on each flank. Mice were fed with a standard chow diet containing complete amino acids (Control diet), the same diet adding serine (High S diet), the same diet lacking serine (-S diet), or a diet lacking both serine and glycine (-SG diet). Diets were purchased from Envigo. Diet formulations are listed in Table S1. The tumor volumes were calculated using the following formula: length × width^2^ × 0.5. At the end of treatment period, all mice were euthanized. Tumors were excised, weighed, and then used for serine content analysis.

To examine the effects of serine synthesis on ΔNp63α-mediated carboplatin resistance in vivo, mice were injected at 8 weeks of age with 5 × 10^5^ NCI-H520 stable cells on each flank. Mice were fed with a diet lacking both serine and glycine. Once the tumor volumes reached about 100 mm^3^, tumor-bearing mice were treated with carboplatin (5 mg/kg, 5 times/week, i.p. injection, 3 weeks) or/and NCT-503 (30 mg/kg, 5 times/week, i.p. injection, 3 weeks). At the end of treatment period, all mice were euthanized, and tumors were excised, weighed, and then fixed, embedded, and sectioned. Tumor sections were subjected to IHC staining for Ki67 and Cleaved Caspase 3. As the animal study was exploratory, no statistical tests were applied to determine adequate sample size and no mice were excluded from the analysis.

### Immunohistochemistry (IHC)

IHC analyses were performed as previously described [37, 41, 42]. Human lung adenocarcinoma (HLugA030PG02, *n* = 30) and lung squamous cell carcinoma (OD-CT-RsLug02-004, *n* = 32) tissue microarrays, were obtained from Shanghai Outdo Biotech (Shanghai, China). The microarrays were used to assess the expression of enzymes or transporters in serine metabolism. Antibodies against to PHGDH (Cat. No. 14719-1-AP, 1:100), PSAT1 (Cat. No. 10501-1-AP, 1:100), PSPH (Cat. No. 14513-1-AP, 1:100), SLC1A4 (Cat. No. 13067-2-AP, 1:100), SLC2A1 (Cat. No. 21829-1-AP, 1:100), SLC1A5 (Cat. No. 20350-1-AP, 1:100), GLS (Cat. No. 29519-1-AP, 1:100), SHMT1 (Cat. No. 30192-1-AP, 1:100), SHMT2 (Cat. No. 11099-1-AP, 1:100), Ki67 (Cat. No. 27309-1-AP, 1:100) were purchased from Proteintech (Wuhan, Hubei, China); Antibody against to Cleaved Caspase 3 (#9664, 1:100) was purchased from Cell Signaling Technology (Danvers, MA, USA). The positively stained signals were scanned through a NanoZoomer (Hamamatsu, Japan). The percentage of positive cells was determined by the three authors (L. Y. Deng, X. Yang, and J. L. Zhang) independently. To compare protein expression differences in the different specimen, the AOD (average optical density) score were calculate accordingly previously described [41].

### Human data from publicly database analysis

The TCGA firehose Legacy datasets on the cBioportal platform (cBioPortal, www.cbioportal.org/) were used to analyze the gene expression of serine metabolism enzymes and TP63 in lung cancer. Kaplan–Meier survival curves for lung cancer were generated using data from the KM Plotter database (www.kmplotter.com). The “Hou Lung dataset” from the Oncomine database was used to analyze the Pearson correlation between TP63 and serine biosynthesis enzymes.

### Statistical analysis

Data from cell culture experiments were performed in three independent experiments and are presented as means ± SD. GraphPad prism 8 software was used for all statistical analysis. Unless otherwise indication, differences between groups were assessed using the two-tailed unpaired Student’s *t* test for comparisons between two groups or ANOVA for comparisons involving more than two groups. The homogeneity of clinical data variances was assessed with Levene’s test in SPSS 16 software. The level of significance is indicated as *p < 0.05, **p < 0.01.

## Results

### Elevated serine synthesis enzymes are associated with poor prognosis in lung cancer

Serine metabolism serves as a central metabolic hub in cancer, primarily fueled glucose and glutamine. The glycolytic intermediate 3-phosphoglycerate is channeled into serine biosynthesis via a three-step enzymatic cascade (Fig. 1A). To assess the clinical relevance of serine metabolism in lung cancer, we analyzed TCGA data and observed significant upregulation of key serine synthesis enzymes (PHGDH, PSAT1, PSPH, SLC1A4) and transporters (SLC2A1, SLC1A4, SLC1A5) in tumors compared to normal tissues (Fig. 1B), Critically, high expression of serine synthesis enzymes predicted poor overall survival (Fig. 1C). Subtype analysis revealed that both LUAD and LUSC exhibited elevated mRNA levels of these enzymes compared to normal tissues (Fig. 1D). Notably, serine synthesis enzymes (PHGDH, PSAT1, PSPH) were significantly higher in LUSC than in LUAD (Fig. 1D), a finding corroborated at the protein level by tissue microarray analysis (Fig. 1E, F). These results establish serine synthesis as a hallmark of aggressive lung cancer, particularly in the LUSC subtype.

### Serine confers carboplatin resistance in NSCLC

**Fig. 2:**
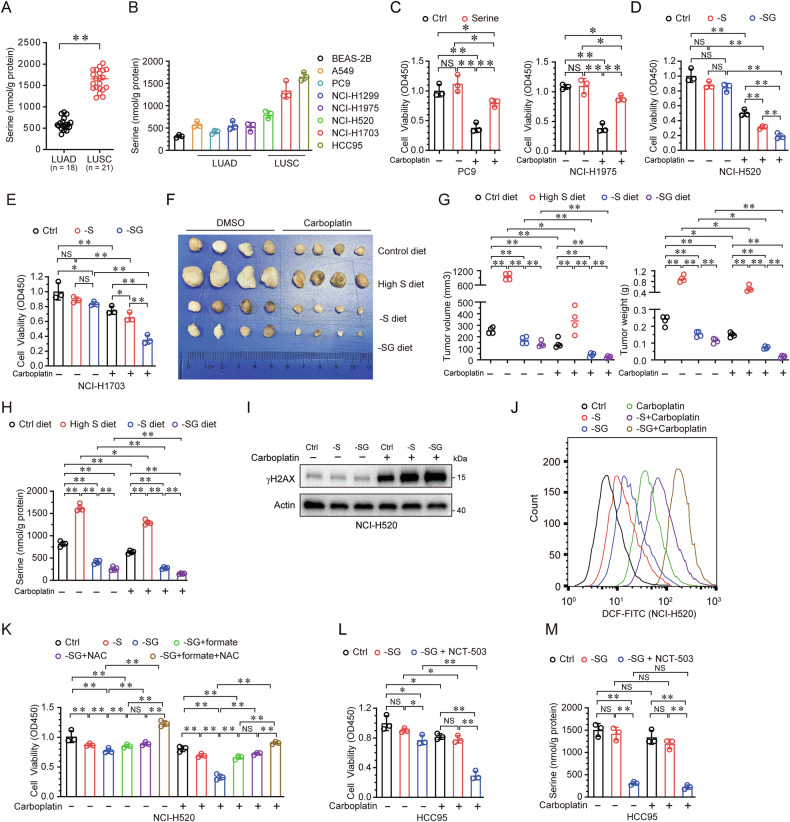
Serine promotes carboplatin resistance in NSCLC. **A** The serine levels were measured in human LUAD and LUSC tissues. **B** The serine levels were measured in cell lines derived from human bronchial, LUAD and LUSC. **C** PC9 or NCI-H1975 cells, cultured in media added with or without serine (1 μM), were treated with carboplatin (100 μM) for 48 h, followed by CCK-8 assay for cell viability. (**D**-**E**) NCI-H520 or NCI-H1703 cells, cultured in media with or without serine (-S) or without both serine and glycine (-SG), were treated with or without carboplatin (100 μM) for 48 h, followed by CCK-8 assay. **F**–**H** Mice were subcutaneously injected with NCI-H520 cells, fed a control diet (23.3 g/kg glycine and 3.5 g/kg serine) or an equivalent diet supplemented with high serine (high S diet, 20 g/kg serine), serine free (-S diet), or both serine and glycine free (-SG diet), were treated with vehicle (DMSO) or carboplatin (5 mg/kg). Representative images of dissected xenografts from the indicated groups at the end of the experiments are shown in (**F**). Tumor volume, tumor weight (**G**) and intratumoural serine levels (**H**) of subcutaneous xenografts were measured. NCI-H520 cells, grown with or without serine (-S) or without both serine and glycine (-SG), were treated with carboplatin (100 μM) for 48 h, followed by immunoblot analysis for γH2AX expression (**I**), or FACS assay for ROS level (**J**). **K** NCI-H520 cells, grown in control (Ctrl), serine deficient (-S), serine and glycine deficient (-SG), -SG + formate (5 μM), -SG + NAC (1 mM) or -SG + formate + NAC media, were treated with or without carboplatin (100 μM) for 48 h, followed by CCK-8 assay. HCC95 cells, grown with or without both serine and glycine (-SG), or combined with or without NCT-503 (5 μM), were treated with or without carboplatin (100 μM), for 48 h, followed by CCK-8 assay (**L**) and measurement for cellular serine level (**M**). *p < 0.05; **p < 0.01; NS not significant.

Given the prominence of serine metabolism in LUSC, we quantified serine levels in clinical samples and cell lines driven from LUAD and LUSC. LUSC tissues and cell lines exhibited significantly higher serine content compared to LUAD counterparts (Fig. 2A, B). Strikingly, cellular serine levels positively correlated with the half maximal inhibitory concentration (IC_50_) of carboplatin across NSCLC cell lines (Fig. 2B, Supplementary Fig. 1A, B), suggesting that serine mediates carboplatin resistance. Further studies demonstrated that exogenous serine supplementation could elevate intracellular serine level and rescue carboplatin-induced cytotoxicity (Supplementary Fig. 1C and Fig. 2C, D). Dual serine/glycine deprivation, rather than serine deprivation alone, synergized with carboplatin in LUSC-derived cells (Fig. 2E), implicating glycine-derived serine in resistance. To determine whether reducing either or both sources of serine affects carboplatin efficacy in vivo, we injected NCI-H520 cells into the flanks of athymic, immunodeficient mice and fed them either complete amino acid-based diet (Ctrl diet), a high-serine diet (High S diet), a serine-deficient diet (-S diet) or a serine and glycine-deficient diet (-SG diet). High-serine diets significantly elevated intratumoral serine level and blunted carboplatin efficacy, while serine-free (-S) or serine/glycine-free (-SG) diets markedly reduced intratumoral serine level and potentiated tumor suppression (Fig. 2F–H).

Serine is a major donor of one-carbon units that are utilized to stimulate nucleotide synthesis [43]. Previous studies have shown that a reduced nucleotide pool and nucleotide imbalances favor DNA damage [44]. To determine whether a DNA damage response occurred under serine-deprived conditions, we tested the presence of phosphorylated histone 2AX (γH2AX), a marker of double-stranded DNA breaks, in NCI-H520 cells. As shown in Fig. 2I, there was minimal accumulation of γH2AX with carboplatin treatment alone; however, a robust increase was observed when serine, or both serine and glycine were removed. Furthermore, carboplatin-induced ROS levels were significantly elevated when serine, or both serine and glycine, were removed (Fig. 2J). Since serine utilization leads to formate production to support nucleotide synthesis and GSH generation to facilitate ROS elimination [43, 45], we reasoned that adding back exogenous formate or clearing the ROS would protect cells from carboplatin-induced cytotoxicity. As shown in Fig. 2K, the addition of formate or N-Acetylcysteine (NAC) strongly recovered cell viability after carboplatin treatment when both serine and glycine were removed. Importantly, the addition of both formate and NAC almost completely rescued cell viability. However, depletion of serine and glycine did not enhance carboplatin cytotoxicity in HCC95 cells, which have high endogenous serine levels (Fig. 2L). Further analysis revealed that the cellular serine content in HCC95 cells decreased slightly when both serine and glycine were removed, suggesting that the serine biosynthesis pathway may maintain endogenous serine levels in these cells (Fig. 2M). Therefore, inhibition serine biosynthesis by NCT503, an inhibitor of PHGDH, markedly enhanced the cytotoxicity of carboplatin when both serine and glycine were removed (Fig. 2L). Taken together, these data indicate that serine promotes carboplatin resistance in NSCLC.

### ΔNp63α regulates serine biosynthesis and carboplatin resistance

**Fig. 3:**
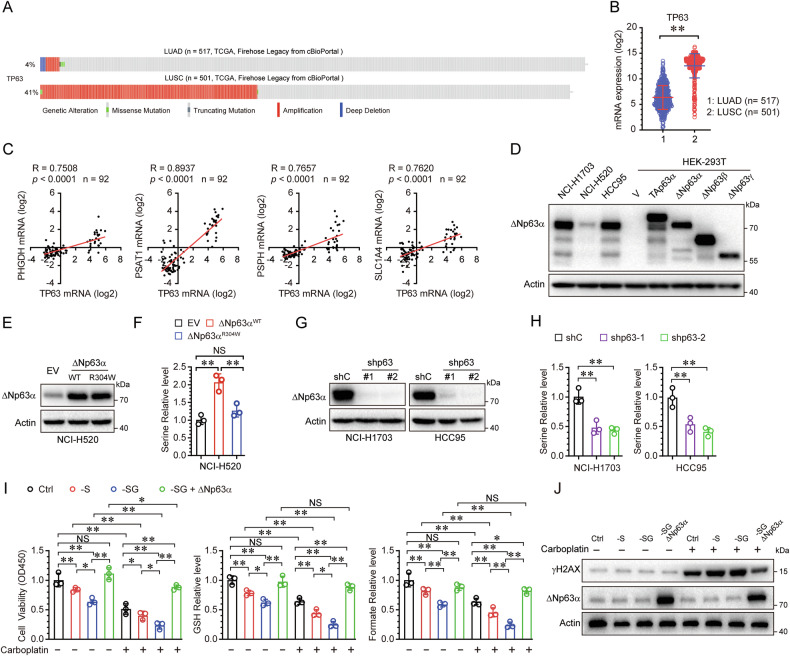
Serine-mediated carboplatin resistance is regulated by ΔNp63α in LUSC. The TCGA Firehose Legacy dataset on the cBioPortal platform was used to analyze TP63 gene alterations (**A**) and mRNA expression (**B**) in LUAD and LUSC. **C** The “Hou Lung dataset” from the Oncomine database was used to analyze the Pearson correlation coefficient (R value) and a two-tailed probability test (p-value) between the mRNA levels of TP63 and serine synthesis enzymes. **D** Whole cell lysates derived from NCI-H1703, NCI-H520, HCC95 cells, and HEK-293T cells transiently transfected with TAp63α, ΔNp63α, ΔNp63β, or ΔNp63γ were subjected to immunoblot analysis. NCI-H520 cells stably expressing ΔNp63α (WT and R304W) were subjected to immunoblot analysis (**E**) and measurement for cellular serine level (**F**). NCI-H1703 and HCC95 cells stably expressing a control shRNA (shC) or two different shRNAs specific for p63 were subjected to immunoblot analysis (**G**) and measurement for cellular serine level (**H**). **I**, **J** NCI-H520 cells stably expressing empty vector (EV) or ΔNp63α were grown in media with or without serine (-S), without serine and glycine (-SG), and treated with or without carboplatin for 48 h, then subjected to CCK-8 assay for cell viability, measurement for GSH and formate levels (**I**), and immunoblot analysis for γH2AX level (**J**). *p < 0.05; **p < 0.01; NS not significant.

Given that serine levels in LUSC are significantly higher than that in LUAD, this suggests that serine biosynthesis might be activated in LUSC. ΔNp63α is a lineage-dependent oncogene in squamous cell carcinoma (SCC) and is often as a diagnosis biomarker for LUSC [46, 47]. The TP63 gene, which encodes the ΔNp63α protein, is frequently amplified at the genomic level (Fig. 3A) and highly expressed (Fig. 3B) in a cohort of LUSC human samples compared to LUAD. To better understand whether there is a mechanistic link between ΔNp63α and serine biosynthesis, we utilized the Oncomine database to analyze the correlation between TP63 mRNA expression and the expression of serine biosynthesis genes. As shown in Fig. 3C, the mRNA levels of serine biosynthesis enzymes (PHGDH, PSAT1, PSPH and SLC1A4) was positively correlated with TP63 mRNA levels. We assessed ΔNp63α expression in LUSC cell lines, selecting one cell line with low (NCI-H520) and two cell lines with elevated (NCI-H1703 and HCC95) endogenous levels of ΔNp63α expression (Fig. 3D). Next, we investigated whether ΔNp63α affects serine biosynthesis. Ectopic expression of wild-type ΔNp63α, but not the transactivation-defective mutant (R304W), markedly increased intracellular serine level (Fig. 3E, F). Conversely, knockdown of ΔNp63α significantly decreased intracellular serine level (Fig. 3G, H). Ectopic expression of ΔNp63α partially restored cell viability, as well as the levels of GSH and formate, which were inhibited by carboplatin under serine and glycine deprived (Fig. 3I). Moreover, ΔNp63α also reduced carboplatin-induced γH2AX accumulation under serine and glycine deprived (Fig. 3J). These data indicate that ΔNp63α regulates serine biosynthesis to promote carboplatin resistance in LUSC.

### ΔNp63α is a direct transcriptional inducer of serine synthesis enzymes

**Fig. 4:**
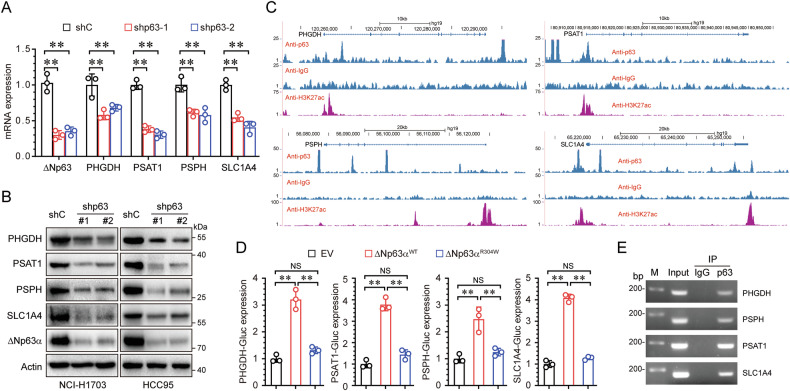
ΔNp63α directly transactivates gene expression of serine synthesis enzymes. **A** HCC95 cells stably expressing a control shRNA (shC) or two different shRNAs specific for p63 were subjected to QPCR analysis. **B** NCI-H1703 or HCC95 cells stably expressing a control shRNA (shC) or two different shRNAs specific for p63 were subjected to immunoblot analysis. **C** p63 chromatin immunoprecipitation (ChIP) sequencing data from human keratinocytes (GEO accession number GSE32061) identifies putative p63-binding sites within the promoter or enhancer regions of PHGDH, PSAT1, PSPH, and SLC1A4 genes. **D** HEK-293T cells were co-transfected with reporter plasmids (PHGDH-Gluc, PSAT1-Gluc, PSPH-Gluc, or SLC1A4) and ΔNp63α (WT or R304W) expression plasmid. Gluc and SEAP activities in the media were measured at 48 h post-transfection. **E** ChIP assays using ΔNp63 antibody or normal rabbit IgG were performed in HCC95 cells. *p < 0.05; **p < 0.01; NS not significant.

To elucidate the molecular mechanism by which ΔNp63α regulates serine synthesis, we knocked down ΔNp63α expression. As shown in Fig. 4A, B, knockdown of ΔNp63α resulted in a significantly decrease in both mRNA and protein levels of PHGDH, PSAT1, PSPH and SLC1A4. Since ΔNp63α is a transcription factor, we hypothesized that ΔNp63α may act as a direct transcriptional activator of serine synthesis enzyme gene expression. To test this hypothesis, we examined available chromatin immunoprecipitation sequencing (ChIP-seq) data in human keratinocytes. These data revealed ΔNp63α binds to the promoters or enhancers of PHGDH, PSAT1, PSPH and SLC1A4, which correspond to open chromatin regions, as indicated by H3K27 acetylation (H3K27ac) (Fig. 4C). Luciferase reporter assays showed that ectopic expression of ΔNp63α, but not the transactivation defective mutant (R304W), promoted reporter activity of PHGDH-Gluc, PSAT1-Gluc, PSPH-Gluc, SLC1A4-Gluc (Fig. 4D). Furthermore, ChIP assays demonstrated that ΔNp63α directly binds to the gene promoters or enhancers of PHGDH, PSAT1, PSPH and SLC1A4 (Fig. 4E). Taken together, these results demonstrate that ΔNp63α directly transactivates the gene expression of serine synthesis enzymes.

It has reported that ATF4 transcriptionally activates serine biosynthetic genes in response to serine starvation in NSCLC cells [20]. NRF2 also regulates serine biosynthesis via ATF4 in NSCLC [21]. Our previous study demonstrated that ΔNp63α promotes radioresistance in ESCC by stabilizing NRF2 protein levels [39]. To investigate whether ΔNp63α controls serine biosynthesis via ATF4 or NRF2, we knocked down ΔNp63α and measured the expression of ATF4 and NRF2. Knockdown of ΔNp63α resulted in a significant downregulation of ATF4 and NRF2 protein levels (Supplementary Fig. 2A) and a reduction in serine and GSH levels (Supplementary Fig. 2B). We then restored ATF4 or NRF2 expression in ΔNp63α-knockdown HCC95 cells, and found that ATF4, but not NRF2, largely rescued serine level (Supplementary Fig. 2B). Restoration of NRF2 expression in ΔNp63α-knockdown HCC95 cells significantly upregulated GSH levels, but had no effect on serine levels. Additionally, knockdown of ATF4 expression in ΔNp63α-stably expressed NCI-H520 cells, and found that ATF4 knockdown did not alter ΔNp63α-mediated serine elevated (Supplementary Fig. 2C, D), indicate that ΔNp63α regulates serine biosynthesis in a ATF4 independent manner. Collectively, ΔNp63α regulates serine synthesis in a ATF4 independent manner, while ΔNp63α regulates GSH synthesis in a NRF2 dependent manner.

### Targeting serine synthesis inhibits ΔNp63α-mediated carboplatin resistance

**Fig. 5:**
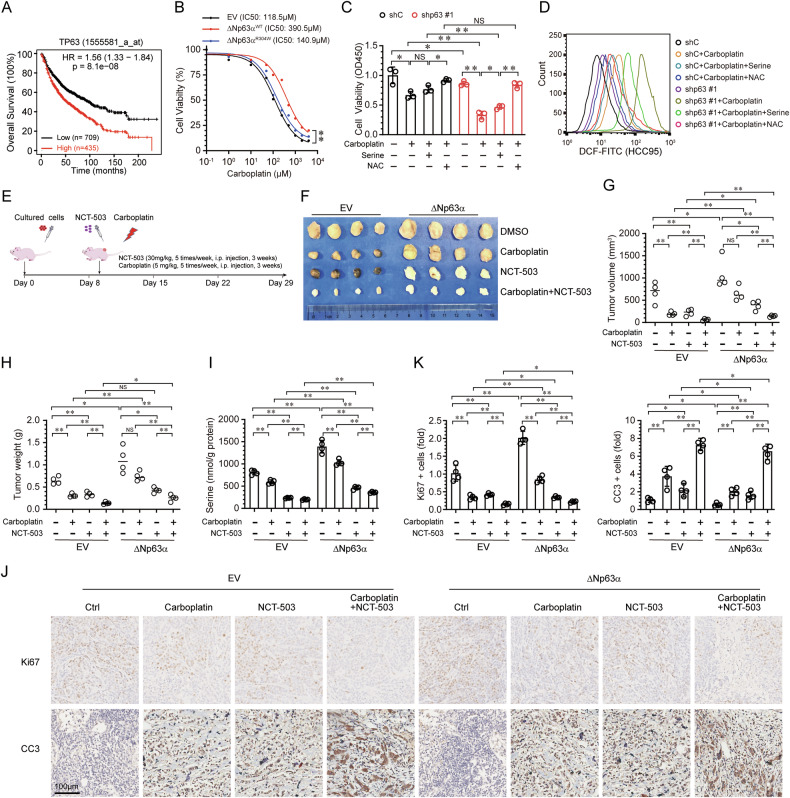
Targeting serine synthesis inhibits ΔNp63α-mediated carboplatin resistance. **A** Overall survival plots of lung cancer in Kaplan–Meier Plotter stratified by TP63 mRNA expression. **B** NCI-H520 cells stably expressing ΔNp63α (WT and R304W) were treated with an indicated dose of carboplatin for 48 h prior to CCK-8 analysis. HCC95 cells stably expressing a control shRNA (shC) or shRNA specific for p63 (shp63 #1) were grown in normal RPMI-1640 media added with or without serine (1 μM) or NAC (1 mM), then treated with or without carboplatin (100 μM) for 48 h, followed by CCK-8 assay for cell viability (**C**) and FACS assay for ROS level (**D**). **E**−**I** Mice were subcutaneously injected with NCI-H520 cells, which stably expressed empty vector (EV) or ΔNp63α, fed with an equivalent diet lacking both serine and glycine, were treated with vehicle (DMSO), or carboplatin (5 mg/kg) combined with NCT-503 (30 mg/kg). Schematic description of the animal experimental design was shown in (**E**). Representative images of dissected xenografts from the indicated groups at the end of the experiments are shown in (**F**). Tumor volume (**G**), tumor weight (**H**) and intratumoural serine level (**I**) of subcutaneous xenografts were measured. **J** Representative immunohistochemistry images of Ki67 and cleaved caspase 3 (CC3) in the indicated group. **K** The protein levels of Ki67 and CC3 were quantified by average optical density (AOD).

Furthermore, we investigate the effect of ΔNp63α on lung cancer progression. As shown in Fig. 5A, high expression of TP63 mRNA was predictive of poor survival in lung cancer. Given that ΔNp63α is predominantly expressed in LUSC and regulates serine biosynthesis, we next examined whether ΔNp63α confers carboplatin resistance through serine. Ectopic expression of wild-type ΔNp63α, but not the mutant (R304W), markedly increased cell survival under carboplatin treatment (Fig. 5B). Knockdown of ΔNp63α significantly enhanced the cytotoxicity of carboplatin (Fig. 5C). Additionally, carboplatin-induced ROS was significantly elevated in ΔNp63α-knockdown HCC95 cells (Fig. 5D). Clearance of the ROS by NAC dramatically increased cell survival (Fig. 5C). The supplementation of serine to ΔNp63α-knockdown HCC95 cells significantly elevated cell viability under carboplatin treatment, although the effect was less pronounced than NAC (Fig. 5C). These data indicate that serine contributes to ΔNp63α-mediated carboplatin resistance.

To examine the effects of serine synthesis on ΔNp63α-mediated carboplatin resistance, we overexpressed ΔNp63α in cells and assessed their sensitivity to carboplatin under full serine/glycine availability, or serine/glycine deprivation, with or without NCT-503. As shown in Supplementary Fig. 3, ectopic expression of ΔNp63α enhanced cell viability. Carboplatin significantly reduced cell viability, especially under serine/glycine deprivation, while overexpression of ΔNp63α increased cell viability under carboplatin treatment under serine/glycine deprivation. Notably, carboplatin combined with PHGDH inhibition significantly suppressed ΔNp63α-mediated cell viability, indicating that inhibition of serine synthesis can counteract ΔNp63α-mediated carboplatin resistance.

To further examine the effects of serine synthesis on ΔNp63α-mediated carboplatin resistance in vivo, we generated xenograft models by subcutaneously injecting transfected LUSC cells into nude mice, followed by treatment with carboplatin and/or NCT-503, an inhibitor of PHGDH (Fig. 5E). As shown in Fig. 5F−I, ectopic expression of ΔNp63α enhanced tumor growth, weight and intratumoral serine level. Carboplatin significantly reduced tumor growth and weight, while overexpression of ΔNp63α promoted tumor growth despite carboplatin treatment. Notably, carboplatin combined with PHGDH inhibition significantly suppressed ΔNp63α-mediated tumor growth, indicating that inhibition of serine synthesis can counteract ΔNp63α-mediated carboplatin resistance. Furthermore, immunohistochemistry assays showed that ΔNp63α increased Ki67+ cells and decreased cleaved Caspase-3+ (CC3) apoptotic cells compared with the empty vector (EV) group after carboplatin treatment (Fig. 5J, K). However, inhibition of PHGDH, when combined with carboplatin treatment, significantly reduced Ki67+ cells and increased CC3+ apoptotic cells. These results indicate that inhibition of serine synthesis, combined with restriction of exogenous serine sources, can counteract ΔNp63α-mediated carboplatin resistance in NSCLC.

## Discussion

Serine metabolism represents a critical metabolic vulnerability in cancer, yet its role in lung cancer biology, particularly in mediating therapeutic resistance, remains incompletely understood. Serine metabolism is differently reprogrammed in LUAD and LUSC, the major subtypes of NSCLC [48]. It has been reported that NKX2-1, lineage-specific oncogene in LUAD, driven serine/glycine synthesis, which generates nucleotides and redox molecules, and is associated with an altered cellular lipidome and methylome [49]. Our study elucidates the mechanistic link between ΔNp63α-driven serine biosynthesis and carboplatin resistance in NSCLC, with specific relevance to the LUSC subtype. We demonstrate that serine synthesis enzymes (PHGDH, PSAT1, PSPH, SLC1A4) are overexpressed in lung cancer, correlating with poor prognosis and elevated serine levels in LUSC compared to LUAD. Serine confers carboplatin resistance by fueling nucleotide synthesis, maintaining redox homeostasis, and mitigating DNA damage. Critically, we identify that ΔNp63α transcriptionally activates these enzymes, creating a metabolic dependency that enables tumor cells to evade carboplatin-induced cytotoxicity. The therapeutic potential of this axis is underscored by our finding that dual inhibition of endogenous serine synthesis (via PHGDH inhibitors) and exogenous serine uptake synergistically overcomes ΔNp63α-mediated resistance, providing a novel strategy for this therapeutically challenging subtype.

Serine functions as an oncogenesis-supportive metabolite through its indispensable roles in macromolecule synthesis, redox homeostasis, and epigenetic regulation. As a primary carbon source, serine fuels nucleotide synthesis for DNA/RNA production, provides precursors for lipid and protein biosynthesis, and supplies one-carbon units for methylation reactions essential for epigenetic control [50]. Equally critical is serine’s contribution to redox balance through glutathione (GSH) and NADPH generation, which scavenge reactive oxygen species (ROS) and mitigate oxidative stress, a key mechanism of chemotherapy resistance [13, 51, 52]. These interconnected functions explain why dietary serine/glycine restriction can suppress tumor growth and sensitize cancers to therapy [25]. However, tumors often evade such interventions by upregulating de novo serine synthesis, exemplified by *PHGDH* amplification in melanoma and breast cancer [23], or oncogene-driven SSP activation (e.g., KRAS, MYC) [30, 44, 53]. Our work confirms that dual suppression of exogenous uptake and endogenous synthesis is essential to potentiate carboplatin efficacy in NSCLC, a strategy particularly effective against ΔNp63α-driven LUSC.

p63 together with p73, belongs to the p53 family of transcription factors. Loss of wild-type *TP53* (WT *TP53*) and certain common mutations in *TP53* (R175H and R273H, but not R248W) render cells dependent on exogenous serine [17]. Furthermore, p53 activation suppresses PHGDH expression by binding to its promoter in p53-WT melanoma cell lines [28]. It has been reported that glutaminolysis plays a critical role in sustaining tumor cell survival upon glucose deprivation [54]. p73 indirectly modulates serine flux via the regulation of glutaminolysis [55]. Therefore, serine serves as a critical carbon source and sustains tumor cell survival under glucose deprivation. The *TP63* gene generates two major isoforms, TAp63 and N-terminal truncated ΔNp63, using different promoters. Alternative splicing at the C terminus further produces different isoforms (α, β, γ, δ, ε) [56]. Accumulation evidence demonstrates that p63 links cell metabolism, including glucose metabolism, glutamine, lipid metabolism, nucleotide metabolism [57]. In this study, we found ΔNp63α directly binds and transactivates the promoters or enhancers of PHGDH, PSAT1, PSPH, and SLC1A4. These results are broadly consistent with reports that p63 regulates serine and one carbon metabolism in head and neck squamous cell carcinoma (HNSCC) [57]. It has been reported that ΔNp63α plays an important role in drug resistance by increasing the transcription of AKT1, EGFR, 14-3-3δ, WIP1, or by downregulating CD95, BAX [58]. Our previous study demonstrated that ΔNp63α promotes radioresistance in esophageal squamous cell carcinoma (ESCC) by stabilizing NRF2 protein to clear ROS [39]. In this study, we demonstrated that ΔNp63α regulates GSH, which scavenges ROS and is generated from serine and glycine, in a NRF2-dependent manner. Notably, it has been reported that ΔNp63α inhibits oxidative stress-induced cell death by direct transactivating the expression of GCLC or GPX2 to catalyze GSH synthesis in a NRF2 independent manner [59, 60]. These findings position ΔNp63α as a central integrator of metabolic rewiring and therapy resistance, redefining it as a “metabolic oncogene” to LUSC.

Our preclinical data validate the metabolic co-targeting as a viable approach to combat carboplatin resistance in ΔNp63α-driven LUSC. Combining PHGDH inhibitors (e.g., NCT-503) with serine/glycine restriction exacerbates DNA damage and ROS accumulation, ultimately enhancing tumor suppression in vivo. This combined approach overcomes the limitations of single-pathway inhibition by addressing the metabolic plasticity that often undermines targeted therapies. For clinical translation, we propose stratifying LUSC patients based on ΔNp63α/SSP enzyme expression to identify candidates for serine metabolism-targeted therapy. Furthermore, the developing of ΔNp63α degraders could disrupt its transcriptional control over SSP.

In summary, our work delineates a ΔNp63α-SSP axis as a key driver of carboplatin resistance in LUSC. By transcriptionally activating serine synthesis enzymes, ΔNp63α establishes a metabolic dependency that sustains nucleotide synthesis, redox balance, and DNA damage tolerance. Dual targeting of serine availability, through PHGDH inhibition and dietary restriction, represents a promising therapeutic paradigm to overcome resistance in this aggressive subtype. These findings underscore the importance of lineage-specific metabolic vulnerabilities in advancing precision oncology for lung cancer.

### Reporting summary

Further information on research design is available in the Nature Research Reporting Summary linked to this article.

## Supplementary information

Supplementary Figures and Table

Reporting Summary

Original Western blots

## Data Availability

The data analyzed during this study are included in this manuscript.
