# Supplementary material for: ΔNp63α drives serine synthesis to promote carboplatin resistance in NSCLC
Source: Cell Death Dis. 2026 Feb 17;17(1):227. doi: 10.1038/s41419-026-08497-4 (PMC12920890; doi:10.1038/s41419-026-08497-4)
Supplement: Supplementary file 1 — Supplementary Figures and Table [file 41419_2026_8497_MOESM1_ESM.docx]

**Supplementary Figures**


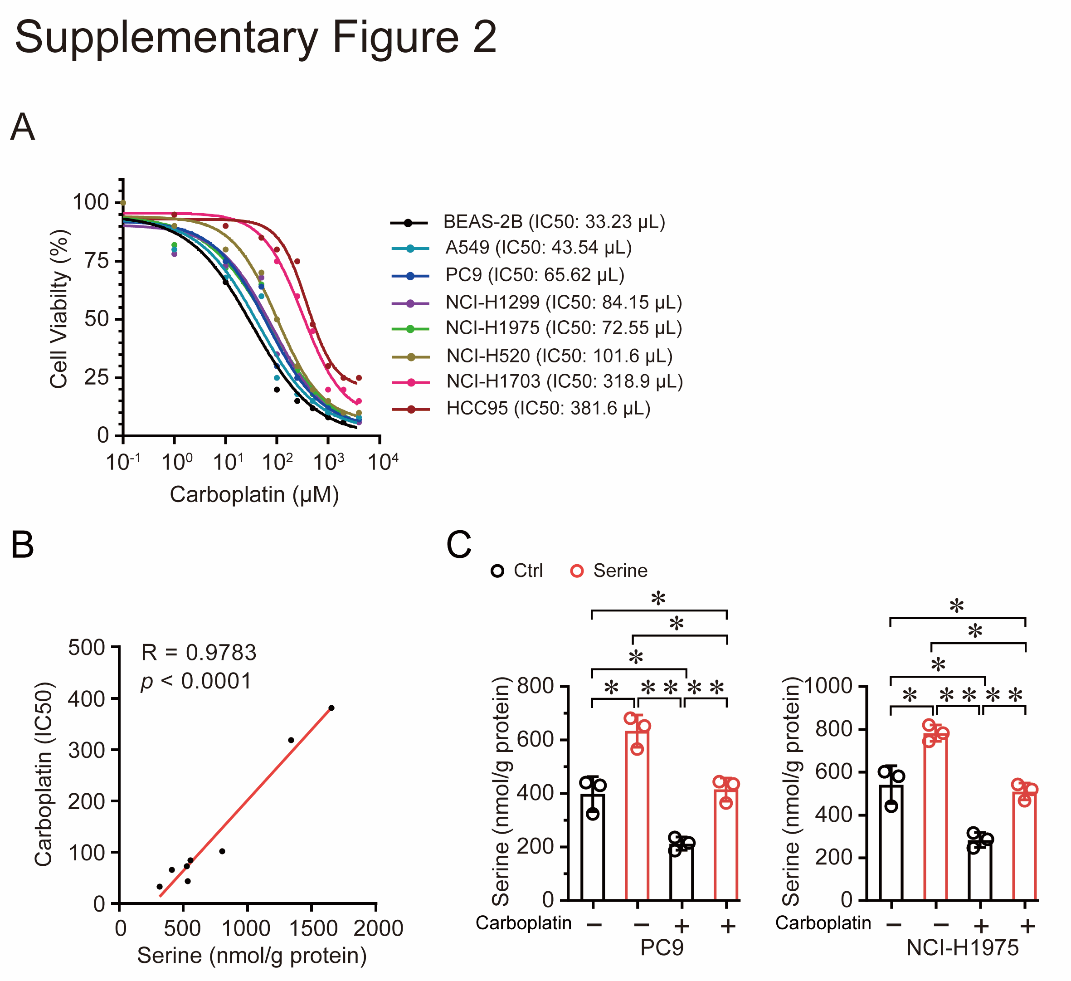


**Supplementary Figure 1**

(A) Cells were treated with the indicated dose of carboplatin for 48 hours prior to CCK-8 assay.

(B) The relationship of serine levels and IC_50_ values of carboplatin for lung cancer cell lines were analyzed using the Pearson correlation coefficient (R value) and a two-tailed probability test (p-value) based on Figure 2B and Supplementary Figure 2A.

(C) PC9 or NCI-H1975 cells, cultured in media added with or without serine (1μM), were treated with carboplatin (100 μM) for 48 hours, followed by measurement for cellular serine level.


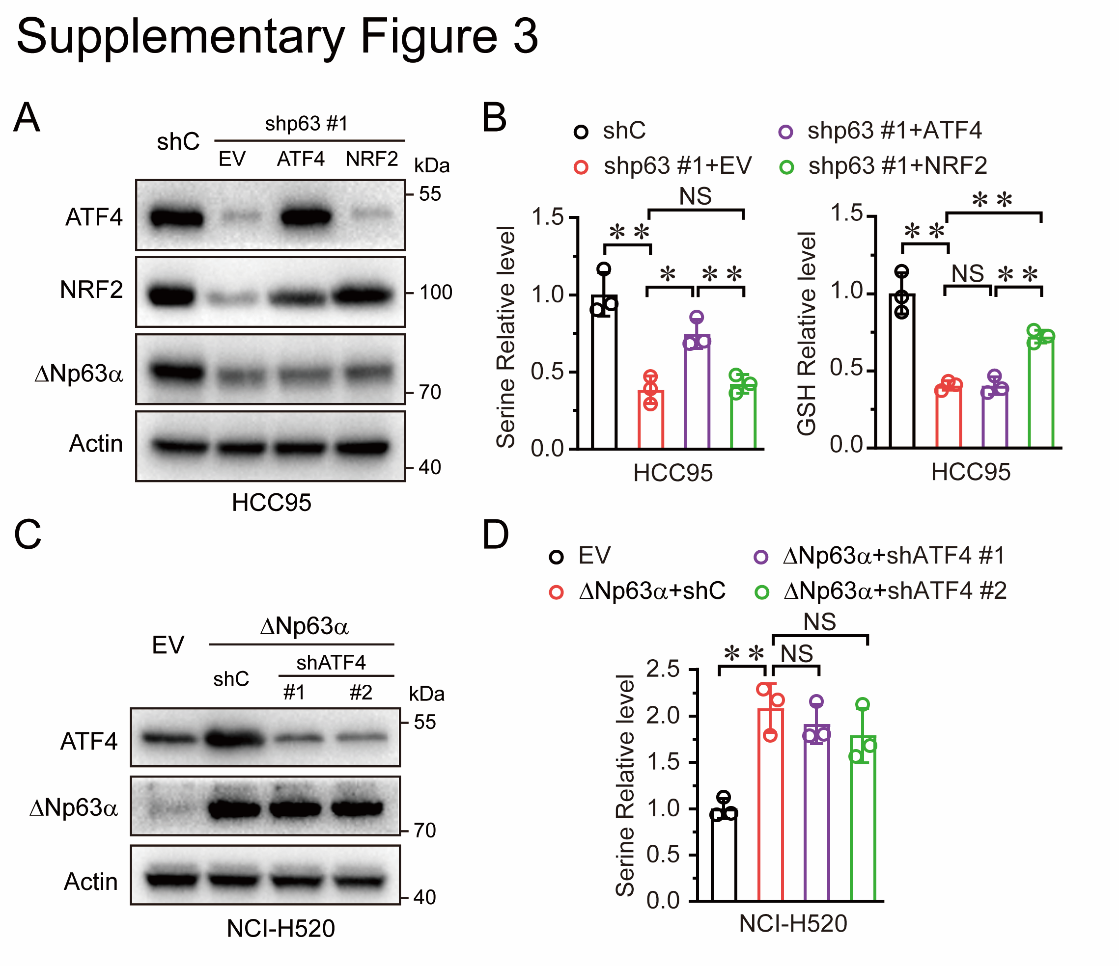


**Supplementary Figure 2**

(A-B) HCC95 cells stably expressing a control shRNA (shC) or shRNA specific for p63 (shp63 #1) were infected with lentivirus expressing ATF4, NRF2, or empty vector (EV), then subjected to immunoblot analysis (A), and measurement for cellular serine and GSH levels (B).

(C-D) NCI-H520 cells stably expressing empty vector (EV) or ΔNp63α were infected with lentivirus expressing a control shRNA (shC) or two different shRNAs specific for ATF4, and then subjected to immunoblot analysis (C), and measurement for cellular serine level (D).


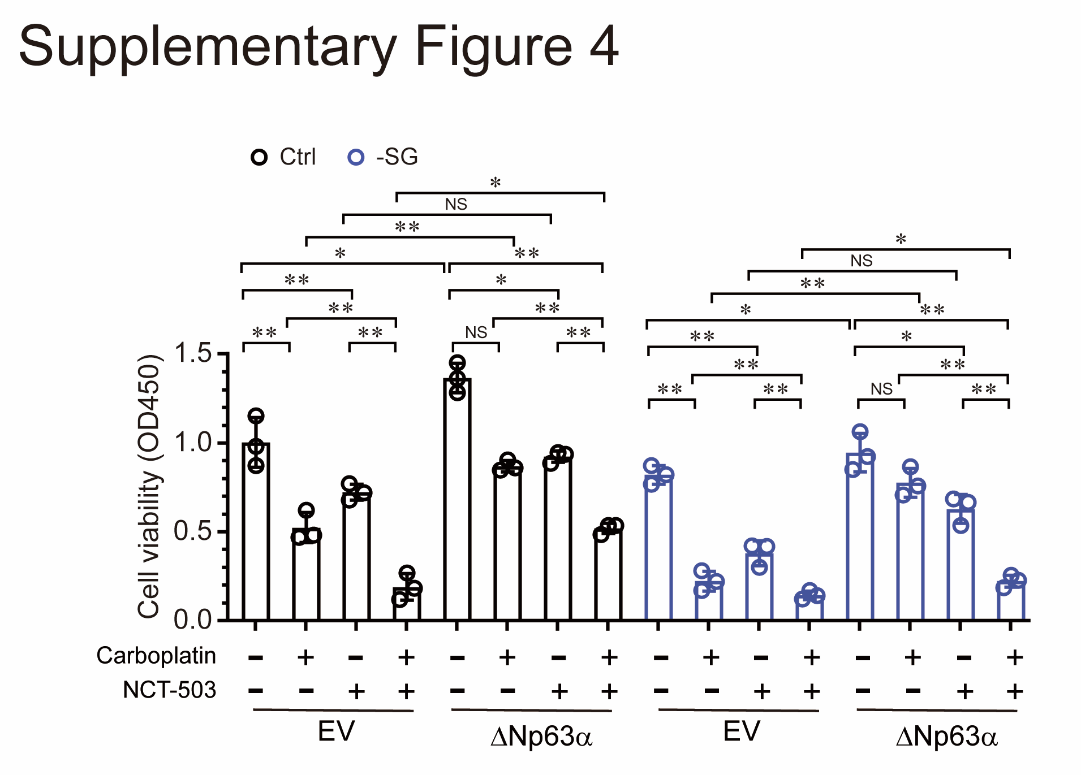


**Supplementary Figure 3**

NCI-H520 cells stably expressing empty vector (EV) or ΔNp63α, were grown in media with or without serine and glycine (-SG), or combined with or without NCT-503 (5 μM), and then treated with or without carboplatin (100 μM) for 48h, followed by CCK8 assay for cell viability.

**Table S1.** Nutrient composition of diets.

| Nutrient | Control diet (g/kg) | High serine diet (g/kg) | Serine free diet (g/kg) | Serine and glycine free diet (g/kg) |
| --- | --- | --- | --- | --- |
| L-Alanine | 3.5 | 3.5 | 3.5 | 3.5 |
| L-Arginine HCl | 12.1 | 12.1 | 12.1 | 12.1 |
| L-Asparagine | 6.0 | 6.0 | 6.0 | 6.0 |
| L-Aspartic Acid | 3.5 | 3.5 | 3.5 | 3.5 |
| L-Cystine | 3.5 | 3.5 | 3.5 | 3.5 |
| L-Glutamic Acid | 40.0 | 40.0 | 40.0 | 40.0 |
| Glycine | 23.3 | 23.3 | 23.3 | 0 |
| L-Histidine HCl, monohydrate | 4.5 | 4.5 | 4.5 | 4.5 |
| L-Isoleucine | 8.0 | 8.0 | 8.0 | 8.0 |
| L-Leucine | 12.0 | 12.0 | 12.0 | 12.0 |
| L-Lysine HCl | 18.0 | 18.0 | 18.0 | 18.0 |
| L-Methionine | 8.2 | 8.2 | 8.2 | 8.2 |
| L-Phenylalanine | 7.5 | 7.5 | 7.5 | 7.5 |
| L-Proline | 3.5 | 3.5 | 3.5 | 3.5 |
| L-Serine | 3.5 | 20.0 | 0 | 0 |
| L-Threonine | 8.2 | 8.2 | 8.2 | 8.2 |
| L-Tryptophan | 1.8 | 1.8 | 1.8 | 1.8 |
| L-Tyrosine | 5.0 | 5.0 | 5.0 | 5.0 |
| L-Valine | 8.0 | 8.0 | 8.0 | 8.0 |
| Sucrose | 100.0 | 100.0 | 100.0 | 100.0 |
| Corn Starch | 381.18 | 364.68 | 384.68 | 407.98 |
| Maltodextrin | 150.0 | 150.0 | 150.0 | 150.0 |
| Soybean Oil | 80.0 | 80.0 | 80.0 | 80.0 |
| Cellulose | 50.0 | 50.0 | 50.0 | 50.0 |
| Mineral Mix, AIN-93M-MX (94049) | 35.0 | 35.0 | 35.0 | 35.0 |
| Calcium Phosphate, monobasic, monohydrate | 8.2 | 8.2 | 8.2 | 8.2 |
| Vitamin Mix, AIN-93-VX (94047) | 13.0 | 13.0 | 13.0 | 13.0 |
| Choline Bitartrate | 2.5 | 2.5 | 2.5 | 2.5 |
| TBHQ, antioxidant | 0.02 | 0.02 | 0.02 | 0.02 |
