# Supplementary figures and images for: ΔNp63α drives serine synthesis to promote carboplatin resistance in NSCLC
Source: Cell Death Dis. 2026 Feb 17;17(1):227. doi: 10.1038/s41419-026-08497-4 (PMC12920890; doi:10.1038/s41419-026-08497-4)

Figure 2

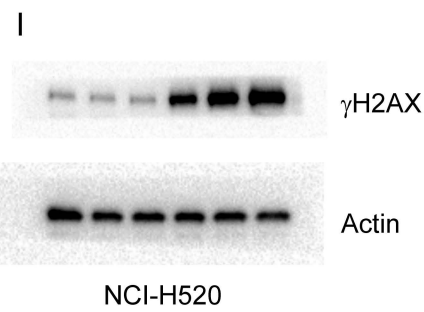

Figure 3

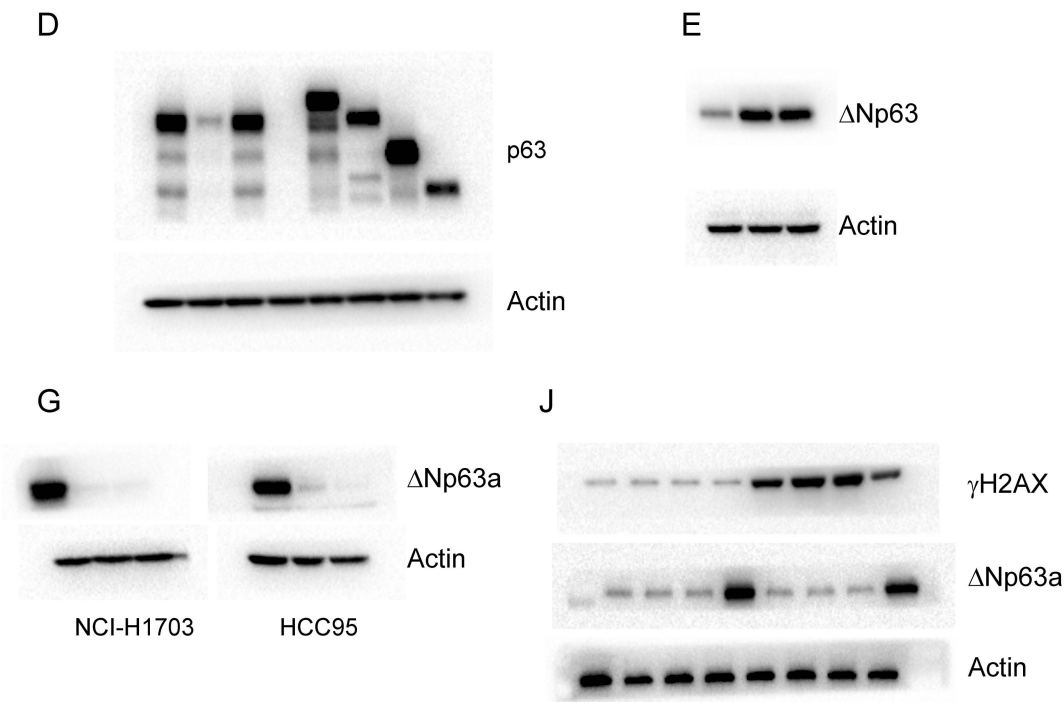

Figure 4

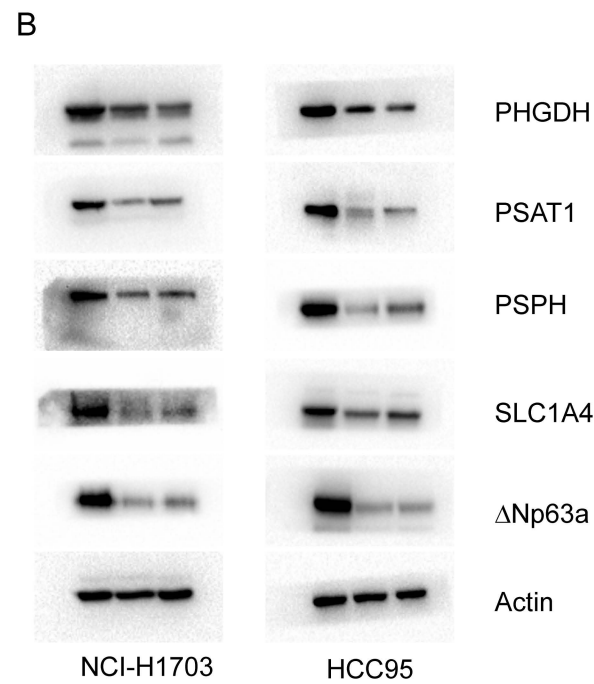

Supplementary Figure 2

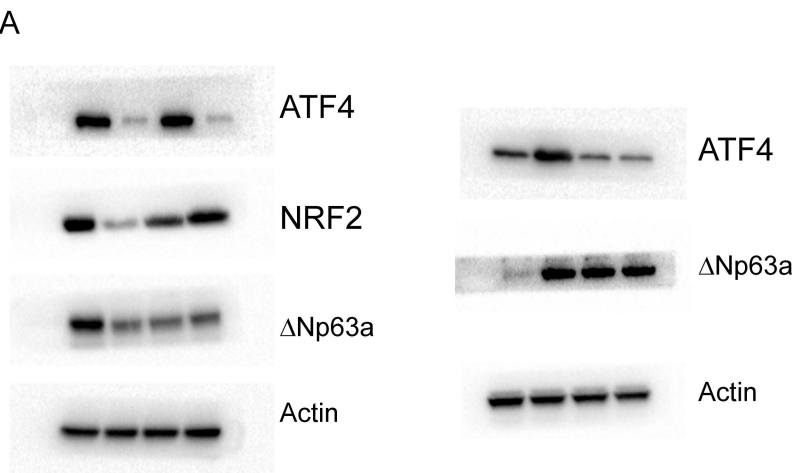

Supplement: Supplementary file 3 — Original Western blots [file 41419_2026_8497_MOESM3_ESM.pdf]
